# Does immunohistochemical staining of P53, Ki 67 and cyclin A accurately predict Wilms tumor recurrence and survival?

**DOI:** 10.1080/2090598X.2022.2058240

**Published:** 2022-04-11

**Authors:** Ahmed M Atwa, Ashraf T Hafez, Mohamed Abdelhameed, Mohamed Dawaba, Adel Nabeeh, Tamer E Helmy

**Affiliations:** a Urology department, Urology and Nephrology Center, Mansoura University, Egypt; b Pathology department, Urology and Nephrology Center, Mansoura University, Egypt

**Keywords:** Wilms’ tumor, relapse, mortality, prediction, immunohistochemistry

## Abstract

**Objective:**

To evaluate whether p53, cyclin A and ki67 immunohistochemical (IHC) assay can be used as predictors for Wilms’ tumor (WT) unfavorable outcomes.

**Methods:**

It is a non-concurrent cohort study including patients who underwent nephrectomy for WT from January 2000 to December 2015 in a tertiary referral center. Over a 5- year follow-up, unfavorable events, including relapse and cancer-specific mortality (CSM), were recorded. P53, cyclin A, and ki67 IHC assay were carried out for formalin-fixed paraffin-embedded WT samples.

**Results:**

After excluding those who did not meet the inclusion criteria, 75 patients were enrolled. Of the patients, 15/75 (20%) experienced WT relapse while 11/75 (14.6%) died of WT over five years. Unfavorable histology (UFH), including prominent blastemal components and anaplasia, was found in 15/75 (20%) children.

Cyclin A immunopositivity was associated with high rates of relapse and CSM. P53 and ki67 positive IHC assay did not show any statistically significant association with unfavorable outcomes. Other risk factors e.g. advanced staging, UFH, extracapsular extension, tumor rupture, lymphadenopathy, and venous thrombosis were not associated with poor prognosis. However, the presence of residual tumors was accompanied by lower survival rates.

**Conclusion:**

Cyclin A IHC assay can be used as a predictor of WT recurrence and CSM. Further studies with prospective patterns and a larger sample size are needed.

**Abbreviations:** WT: Wilms’ tumor, UFH: unfavorable histology, IHC: immunohistochemical, PI: proliferation index, RFS: relapse-free survival, CSS: cancer-specific survival, FH: favorable histology, CSM: cancer-specific mortality, CDK: cyclin-dependent kinase.

## Introduction

Nephroblastoma or Wilms’ tumor (WT) is the most common malignant renal neoplasm during childhood, representing 90% of all malignant pediatric renal tumors and 6% of all pediatric cancers [1]. Tumor staging and unfavorable histology (UFH) are used to give an idea about the prognosis [2]. Advanced staging, diffuse anaplasia, predominant blastemal elements, and lymph node invasion are indicators of poor prognosis [3].

Despite using the previously mentioned parameters, we found that some tumors, which were considered of low risk did not respond to therapy and eventually resulted in mortality. In contrast, other tumors assumed to be of poor prognosis responded dramatically to treatment. Therefore, it is crucial to search for predictors of WT prognosis beside tumor staging and grading. Identification of protein markers regulating cell proliferation can be very useful in predicting WT behavior [4].

P53 is a tumor suppressor gene, and its mutation is identified in various types of human cancer [5]. P53 protein accumulation in certain tumors is associated with tumor aggressiveness [6]. Many studies addressed its association with advanced stages and anaplasia [4,6,7].

Ki67 is a nuclear antigen related to cell proliferation that is present in all phases of the cell cycle G1, S, G2, and mitosis except G0. Ki67 protein is recognized on a wide scale as a proliferation marker that can be useful in predicting the progression of some human cancers because resting cells lack Ki67 [8]. On the other hand, some authors concluded that it may not be a dependable prognostic marker [9].

The Cell cycle progression is under the control of the relative levels of cyclin family members [10]. Cyclin A is a marker for proliferation because its levels rise in the early S-phase and decline in M-phase. The correlation between poor prognosis and increased expression of cyclin A was confirmed in different entities of human cancer such as medulloblastoma and ovarian carcinoma. Recently, Radojevic-Skodric et al deduced that cyclin A overexpression may be associated with the poor prognosis of WT [11].

Ultimately, there should be a cost-effective tool to predict WT progression and recurrence other than genetic studies which are not affordable for the health care system. Nonetheless, there is a paucity of literature regarding the role of immunohistochemical (IHC) staining in expecting WT aggressiveness. The study aims to determine the feasibility of using p53, Ki 67, and cyclin A IHC assay as predictors of WT relapse and survival.

## Material And Methods

### Data collection

In this non-concurrent cohort study, case records of WT were collected from January 2000 to December 2015 at a single tertiary referral center. Exclusion criteria included preoperative chemotherapy (due to areas of necrosis and hemorrhage hindering IHC staining), lost follow-up, and death due to causes other than WT.

After Institutional Review Board approval (MD.19.09.223) and clinical trial registration (NCT04758455), patients’ archived data had been retrieved then reviewed for the inclusion and exclusion criteria. Legible patients were contacted to participate in the study and to sign the informed consent form in line with Good Clinical Practice and the Declaration of Helsinki.

### Immunohistochemical assay

Formalin-fixed paraffin-embedded specimens, obtained from the previously preserved blocks at the pathology laboratory stained with Hematoxylin and Eosin were used for tumor staging and grading. After deparaffinization, rehydration, and blocking endogenous peroxidase activity, antigens were retrieved followed by protein blocking and application of primary antibodies which included (Anti-p53, clone BP-53-12 mouse monoclonal, Genemed, USA), (Anti-ki67, clone GM010, mouse monoclonal, Genemed, USA), (Anti-cyclinA1, clone 28,970,002, rabbit polyclonal, Novus biologicals, USA) and (Power stain 1.0 poly HRP/DAB kit for mouse and rabbit, Genemed, USA).

After that, the slides were incubated for 45 minutes at room temperature in a humid chamber and secondary antibodies were added. Eventually, a streptavidin enzyme label was applied, chromogen was prepared and coverslips were fixed using Canada balsam. Positive staining is indicated by the presence of a brown color.

**Figure 1.**
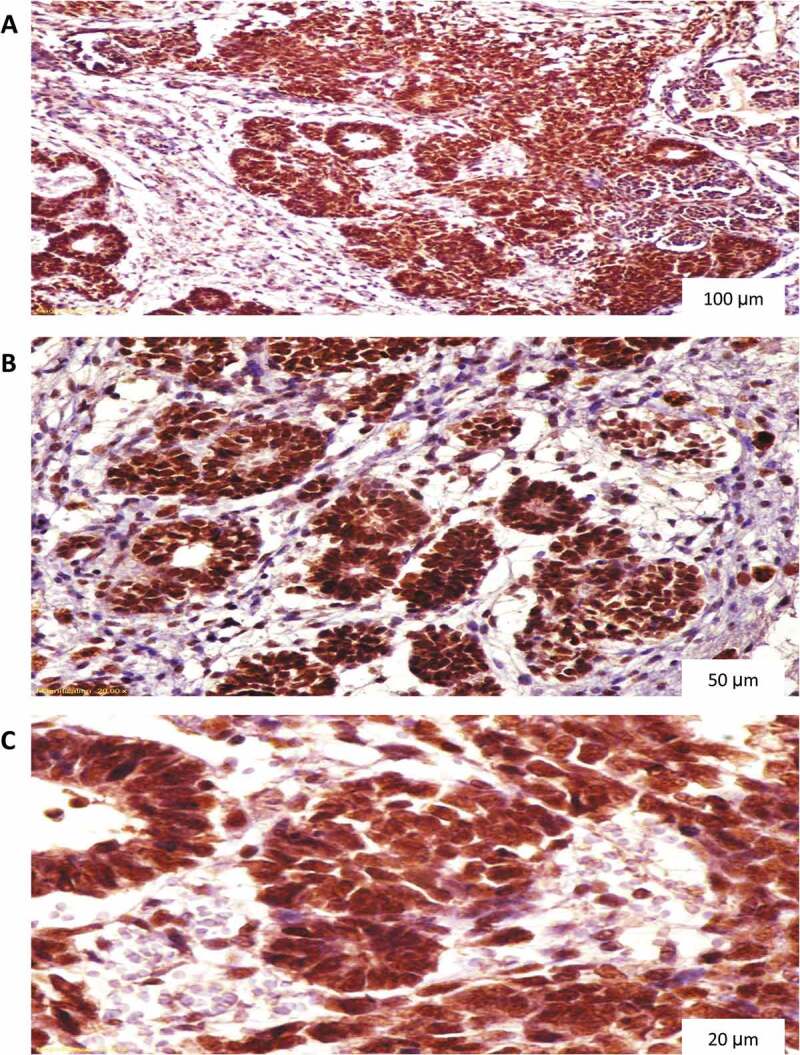
P53 diffuse nuclear staining in nephroblastoma. (A) Magnification X100, (B) Magnification X200, (C) Magnification X400.

**Figure 2.**
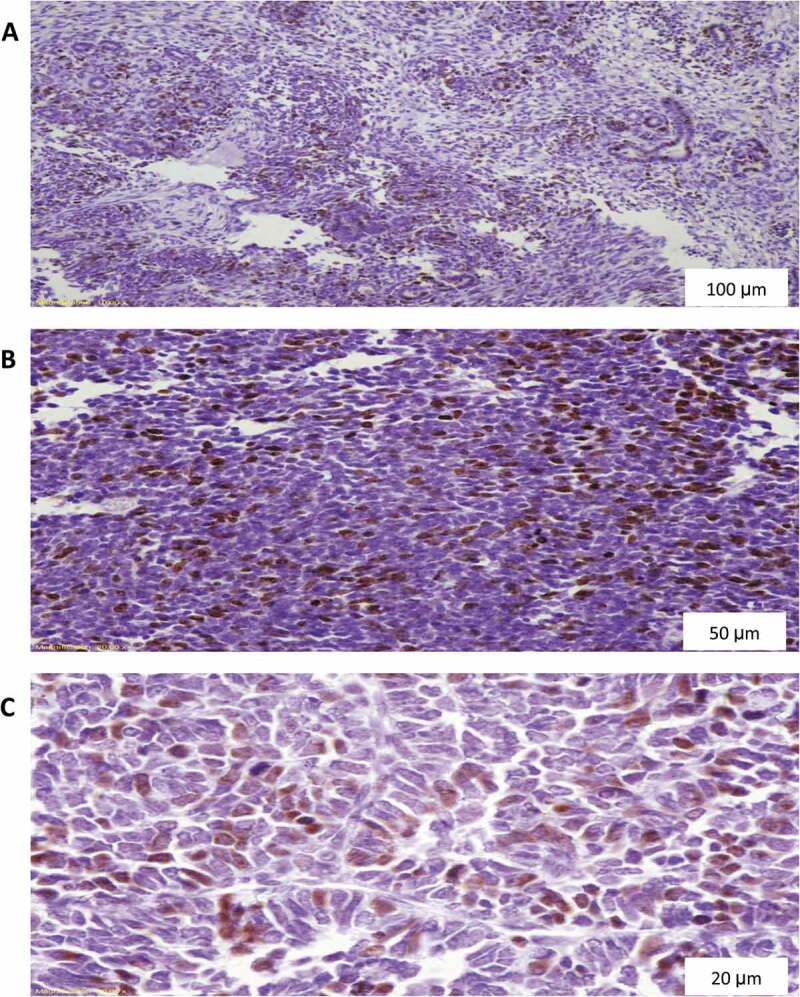
Ki67 diffuse nuclear staining in nephroblastoma. (A) Magnification X100, (B) Magnification X200, (C) Magnification X400.

**Figure 3.**
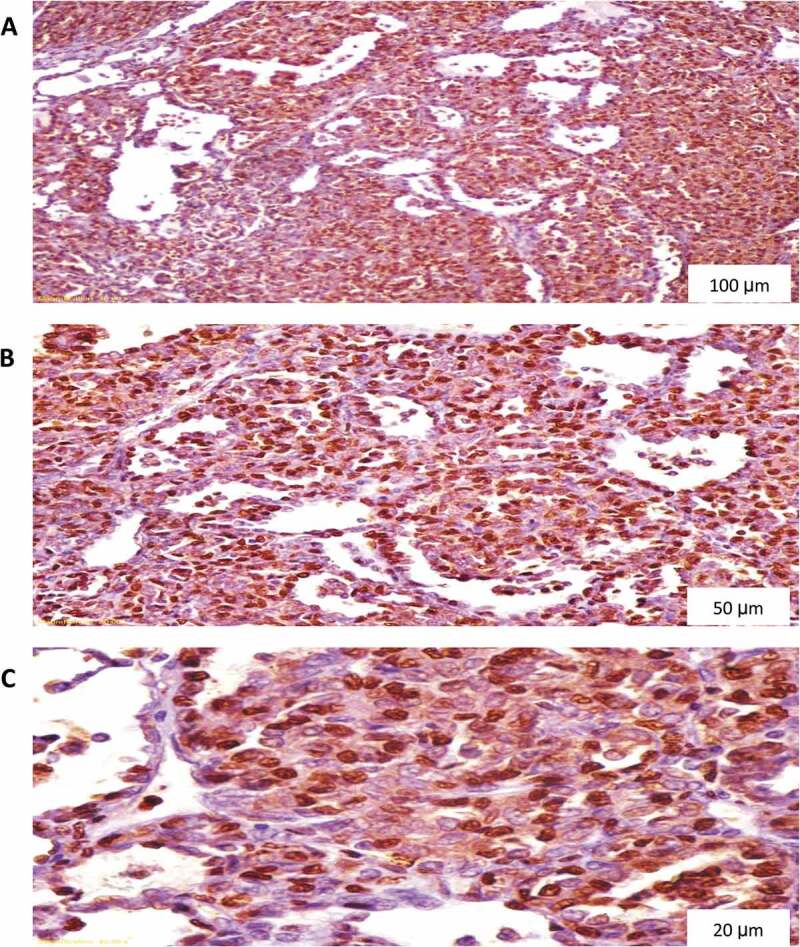
Cyclin A diffuse nuclear staining in nephroblastoma. (A) Magnification X100, (B) Magnification X200, (C) Magnification X400.

All specimens were examined by an experienced histopathologist devoted to the urological practice and blinded to the patients’ clinical findings. P53 and cyclin A staining were scored as 0 (no nuclear staining), 1 (<10% nuclear staining), 2 (10–50% nuclear staining), and 3 (>50% nuclear staining) (Figure 1,2) [12]. Ki67 proliferation index (PI) was graded as low (L), borderline (BL), and high (H) (Figure 3) [13]. For statistical analytic purposes, p53 and cyclin A grades 2 and 3 plus H grade Ki67 were reported as positive (+).

### Statistical analysis

Obtained data were analyzed using the Statistical Package for Social Sciences (SPSS Inc., Chicago, IL, USA, version 26). Frequencies and percentages were used for representing qualitative data while mean and range were used for quantitative data. Normality distribution was tested using Shapiro-Wilks and Kolmogorov-Smirnov tests.

Chi-squared, and Fisher exact tests were used for comparison between groups, as appropriate. Regarding survival analysis, Kaplan–Meier curve was used to calculate the relapse-free survival (RFS) and cancer-specific survival (CSS) while groups were compared using the Log-rank test. RFS was defined as the time from surgery to relapse detection whereas CSS was determined as the interval between surgery and death attributed only to WT. P values of less than 0.05 were considered statistically significant.

## Results

The study cohort started at 81 eligible patients. Of the patients, four lost follow-up and two died of causes other than WT. Therefore, a total of 75 children who underwent nephrectomy for WT, without prior chemotherapy or radiotherapy, were enrolled.

Demographic, clinical, and radiological features were analyzed. The median age of incidence was 36 months. Regarding gender-based distribution, 40/75 (53.3%) cases were boys while 35/75 (46.7%) cases were girls (Table 1). Based on National Wilms Tumor Study (NWTS)-5 staging, tumor stage distribution was as shown; 45/75 (60%) cases in stage I, 8/75 (10.7%) cases in stage II, 10/75 (13.3%) cases in stage III, and 12/75 (16%) cases in stage IV. Favorable histology (FH) was reported in 60/75 (80%) while 15/75 (20%) cases had UFH; 8/75 (10.7%) showed dominant blastemal component and 7/75 (9.3%) demonstrated anaplasia (Table 2).

**Table 1.** Patients’ demographics.

Age (months) Median (Range)	36 (2–216)
Gender N^o^ (%) Boys Girls	40 (53.3) 35 (46.7)
ASA score N^o^ (%) ASA I ASA II ASA III	66 (88) 7 (9.3) 2 (2.7)
BMI (kg/m^2^) Median (Range)	17.73 (3.6–59.2)

**Table 2.** Tumor characteristics.

Tumor size (Maximal diameter in centimeters) Median (Range)		10.50 (3–26)
**Tumor stage** N^o^ (%)		
stage I		45 (60)
stage II		8 (10.7)
stage III		10 (13.3)
stage IV		12 (16)
**Pathology** N^o^ (%)		
Triphasic		50 (66.7)
Blastemal		9 (12)
Epithelial		8 (10.7)
Others: mesoblastic and Biphasic Patterns		8 (10.7)
**Anaplasia** N^o^ (%)		
Focal		4 (5.3)
Diffuse		3 (4)
**UnFavorable histology** N^o^ (%)		15 (20)
**P53 IHC** N^o^ (%)		
0		15 (20)
1		17 (22.7)
2		13 (17.3)
3		30 (40)
**Ki67 IHC** N^o^ (%)		
Low		38 (50.7)
Borderline		12 (16)
High		25 (33.3)
**CyclinA IHC** N^o^ (%)		
0		15 (20)
1		14 (18.7)
2		19 (25.3)
3		27 (36)

**Table 3.** Depicts the percentage of RFS and CSS during follow-up.

Unfavorable outcomes during follow-up	Relapse-free survival (RFS)			Cancer-specific survival (CSS)		
	Total number of cases	Number of relapses	Percentage surviving	Total number of cases (%)	Number of deaths	Percentage surviving (%)
At 12 months	75	0	100	75	0	100
At 24 months	75	0	100	75	0	100
At 36 months	75	0	100	74	1	98.7
At 48 months	72	3	96	72	2	96
At 60 months	69	3	92	71	1	94.7
At 72 months	67	2	89.3	70	1	93.3
At 84 months	66	1	88	70	0	93.3
At 96 months	64	2	85.3	68	2	90.7
At 108 months	63	1	84	66	3	88
At 120 months	60	3	80	64	1	85.3

**Table 4.** The association between Wilms’ tumor relapse and the other variables.

		Number	P of log rank test
Overall		75	
Gender	Boys	40	0.67
	Girls	35	
P53	-	32	0.14
	+	43	
Ki67	-	50	0.12
	+	25	
Cyclin A	-	32	0.01
	+	43	
Tumor side	Right	35	0.47
	Left	40	
Extracapsular extension	No	62	0.26
	Yes	13	
Anaplasia	No	68	0.91
	Yes	7	
Unfavorable histology	No	60	0.13
	Yes	15	
Stage	stage I	45	0.11
	stage II	8	
	stage III	10	
	stage IV	12	
Lymphadenopathy	No	64	0.37
	Yes	11	
Venous thrombus	No	65	0.95
	Yes	10

**Table 5.** The association between cancer specific mortality (CSM) and the other variables.

		Number	P of log rank test
Overall		75	
Gender	Boys	40	0.89
	Girls	35	
P53	-	32	0.57
	+	43	
Ki67	-	50	0.57
	+	25	
Cyclin A	-	32	0.02
	+	43	
Tumor side	Right	35	0.78
	Left	40	
Extracapsular extension	No	62	0.05
	Yes	13	
Anaplasia	No	68	0.73
	Yes	7	
Unfavorable histology	No	60	0.75
	Yes	15	
Stage	stage I	45	0.1
	stage II	8	
	stage III	10	
	stage IV	12	
Lymphadenopathy	No	64	0.75
	Yes	11	
Venous thrombus	No	65	0.47
	Yes	10

**Table 6.** The association between Wilms’ tumor staging and immunohistochemical staining of p53, Ki67 and cyclin A.

	Stage 1 and 2	Stage 3 and 4	P value (Chi-Square)
P53 IHC			
-	22 (41.5%)	10 (45.5%)	0.75
+	31 (58.5%)	12 (54.5%)	
Ki67 IHC			
-	35 (66%)	15 (68.2%)	0.86
+	18 (34%)	7 (31.8%)	
Cyclin A IHC			
-	24 (45.3%)	8 (36.4%)	0.48
+	29 (54.7%)	14 (63.6%)

**Table 7.** The association between unfavorable histology (UFH) and immunohistochemical staining of p53, Ki67 and cyclin A.

	Favorable histology	Unfavorable histology	P value (Chi-Square)
P53 IHC			0.04
-	29 (48.3%)	3 (20%)	
+	31 (51.7%)	12 (80%)	
Ki67 IHC			0.22
-	42 (70%)	8 (53.3%)	
+	18 (30%)	7 (46.7%)	
Cyclin A IHC			0.73
-	25 (41.7%)	7 (46.7%)	
+	35 (58.3%)	8 (53.3%)	

Tumor rupture occurred in 3/75 (4%) patients whereas residual tumor was detected in 2/75 (2.6%) cases. Additionally, venous thrombosis was confirmed upon exploration in 10/75 (13.3%) children. P53, ki67 and cyclin A IHC staining was positive in 43/75 (57.3%), 25/75 (33.3%), and 46/75 (61.3%), respectively.

**Figure 4.**
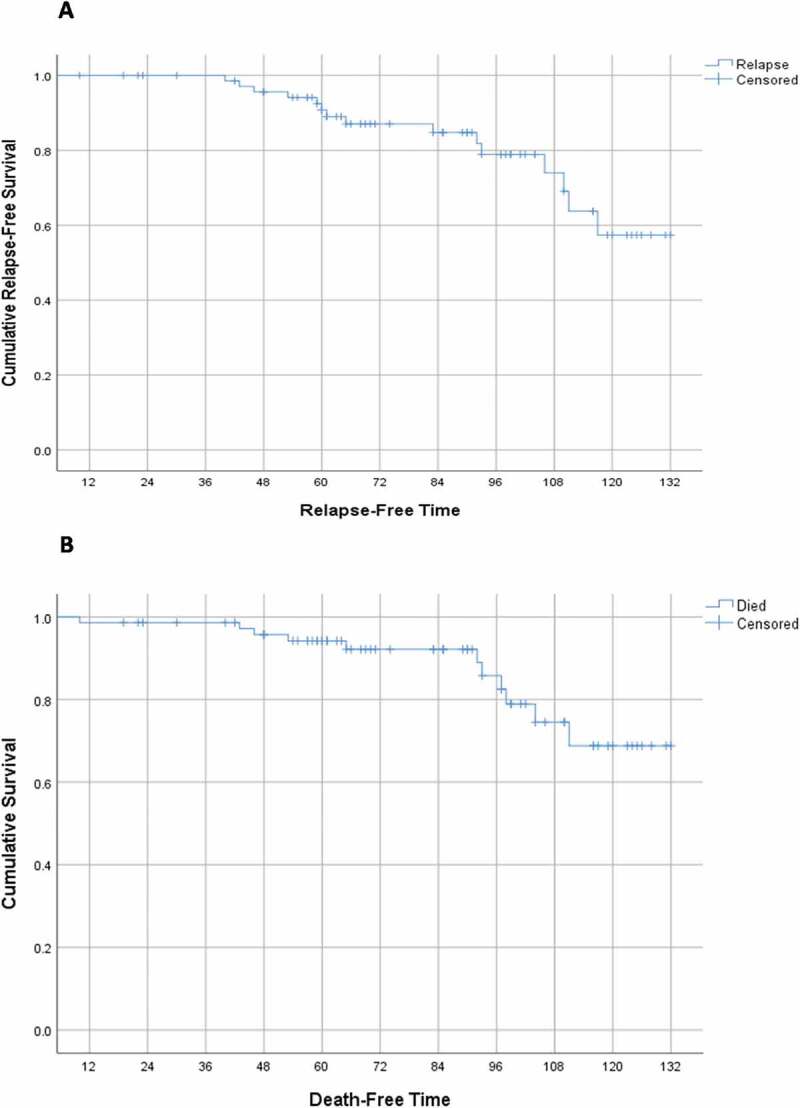
Event-Free survival (EFS) (A) Relapse-free survival (RFS) (B) Cancer-specific survival (CSS).

**Figure 5.**
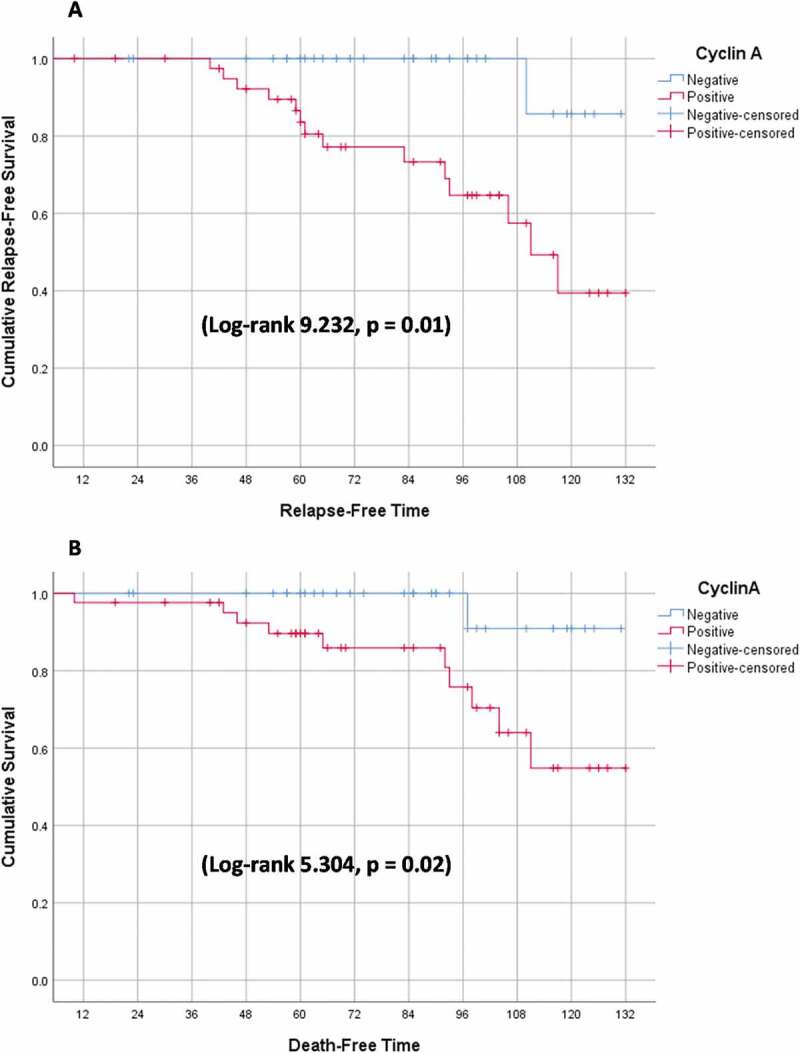
Event-Free survival (EFS) stratified by cyclin A immunohistochemical (IHC) assay (A) Relapse-free survival (RFS) (B) Cancer-specific survival (CSS).

Positive cyclin A staining was associated with higher relapse and mortality rates (P: 0.01 and 0.02, respectively) (Tables 3, 4, 5) (Figure 5). On the other hand, positive P53 and Ki67 IHC staining, UFH, and advanced staging were not associated with poor outcomes (Tables 4-7). Kaplan-Meier curve is depicted in Figure 4.

Multivariate analysis was not applicable for both outcomes because the only predictor of WT relapse was positive cyclin A IHC assay while the second predictor of CSM, which was residual tumor, included only nine cases hindering the performance of binary logistic regression and calculation of the odd’s ratio. Despite using non-parametric data, the mean was used in spite of the median to express the event-free survival (EFS) because the event (relapse or death) occurred in less than 50% of cases.

## Discussion

WT is the most common renal tumor in children and its prognosis relies mainly on tumor stage and UFH [2]. The use of IHC assay to predict WT behavior has attracted the attention of researchers over the last decades, especially in low and medium-income countries [11,14]. Three easily available IHC markers P53, Ki67, and cyclin A were used.

P53 is considered a dam against carcinogenesis because it controls cellular metabolism and survival following exposure to stressful stimuli e.g. hypoxia and DNA damage [15]. P53 overexpression has been studied in many tumors [16]. The results of previous studies on the prognostic value of P53 in WT were inconsistent.

In a meta-analysis conducted by Liu et al, P53 immunopositivity was associated with advanced stage, UFH and WT metastasis [17]. However, P53 expression was not a predictor of recurrence. Even though the advanced stage, and UFH are used as predictors of WT poor prognosis, Huang et al. concluded that P53 expression in FH WT was associated with poor outcomes [17].

Krishna et al. and Das et al. found higher P53 expression in advanced stages of WT [4,18]. On the other hand, Percicote et al. and Madjd et al. found no association between P53 expression and unfavorable outcomes [19,20]. In the current study, P53 positive IHC staining was not a predictor of WT relapse or attributed mortality (P = 0.14 and 0.57, respectively). Furthermore, it was associated with UFH (P = 0.04), but not with advanced stage (P = 0.95) (Tables 5,6).

Ki-67 is one of the nuclear antigens related to cell proliferation. Juric et al deduced that ki67 overexpression can be used for the evaluation of WT proliferative activity, but it may not be a reliable marker to predict WT behavior [9]. Although Das et al did not study the correlation between ki67 expression and WT behavior, they found that ki67 immunopositivity was accompanied by advanced tumor stage [18]. In our study, there were no statistically significant differences between Ki67 positive IHC staining and WT recurrence, CSS, tumor stage, and UFH (P = 0.12, 0.36, 0.86, and 0.22, respectively).

Cyclins are a group of proteins that regulates and controls the cell cycle by binding and stimulating members of the cyclin-dependent kinase (CDK) family. Cyclin A controls the cell cycle and DNA replication via checkpoints [10]. Only one study reviewed the role of cyclin A IHC expression in WT. Radojevic-Skodric et al found that cyclin A expression was high in large-sized tumors (10.5 ± 4.06 cm) and stages 3 and 4 (P = 0.01 and 0.004, respectively). However, there was no statistically significant difference between diffuse anaplasia and other variants (P = 0.27) [11].

In our study, cyclin A expression was not detected in large tumor size, advanced stage, or UFH (P = 0.65, 0.48, and 0.73, respectively). Besides, cyclin A immunopositivity was associated with high recurrence and poor survival rates (P = 0.02).

The present study demonstrated that cyclin A expression is associated with poor prognosis in patients with WT. Nevertheless, P53 and Ki67 overexpression are not associated with high relapse or reduced survival rates. To the best of the authors’ knowledge, this is the first study involving the role of P53, Ki67, and cyclin A in predicting WT behavior in terms of recurrence and survival. The limitations of this study included a relatively small sample size and retrospective nature.

Post hoc power analysis was done using https://www.openepi.com/Power/ Power CC .htm. Using current study results of relapse (as an outcome) of 6.7% versus 93.3% in both cyclin A IHC negative and positive cases; respectively, the study power is 100%. Also using currents study results of death (as an outcome) of 9.1% versus 90.9% in both cyclin A IHC negative and positive cases; respectively, the study power is 100%.

## Conclusion

Cyclin A expression in WT can be used as an indicator of poor prognosis. Nonetheless, p53 and ki67 overexpression were not found to be good predictors of WT unfavorable outcomes. Future prospective studies with a larger number of patients enrolled should be considered.
